# Chronic opioid use modulates human enteric microbiota and intestinal barrier integrity

**DOI:** 10.1080/19490976.2021.1946368

**Published:** 2021-07-27

**Authors:** Angélica Cruz-Lebrón, Ramona Johnson, Claire Mazahery, Zach Troyer, Samira Joussef-Piña, Miguel E. Quiñones-Mateu, Christopher M Strauch, Stanley L. Hazen, Alan D. Levine

**Affiliations:** a Departments of Molecular Biology and Microbiology, Case Western Reserve University, Cleveland, USA; b Department of Pathology, Case Western Reserve University, Cleveland, USA; c Department of Microbiology and Immunology, School of Biomedical Sciences, University of Otago, Dunedin, New Zealand; d Department of Cardiovascular and Metabolic Sciences, Lerner Research Institute, Cleveland, USA; e Departments of Pharmacology, Medicine, and Pediatrics, Case Western Reserve University, Cleveland, USA

**Keywords:** Intestinal permeability, tight junctions, *Akkermansia muciniphila*, short-chain fatty acids, metabolomics, methadone

## Abstract

Over the past three decades the United States has experienced a devastating opioid epidemic. One of the many debilitating side effects of chronic opioid use is opioid-induced bowel dysfunction. We investigated the impact of methadone maintenance treatment (MMT) on the gut microbiome, the gut bacterial metabolite profile, and intestinal barrier integrity. An imbalance in key bacterial communities required for production of short-chain fatty acids (SCFAs), mucus degradation, and maintenance of barrier integrity was identified. Consistent with dysbiosis, levels of fecal SCFAs were reduced in MMT. We demonstrated that metabolites synthesized by *Akkermansia muciniphila* modulate intestinal barrier integrity *in vitro* by strengthening the pore pathway and regulating tight junction protein expression. This study provides essential information about the therapeutic potential of *A. muciniphila* and warrants development of new clinical strategies that aim to normalize the gut microbiome in individuals affected by chronic opioid use.

## Introduction

The opioid epidemic in the United States has been expanding for the past three decades.^1,2^ The CDC reports that of approximately 71,000 drug overdose deaths in 2019, 70% were associated with opioids.^3^ Opioids are used to treat chronic pain clinically but can be highly addictive due to their euphoric effects.^4^ Endogenous opioids, such as enkephalins, endorphins, and dynorphins,^5^ which preferentially bind to their corresponding opioid receptors (ORs), µ-OR, δ-OR, and κ-OR,^5,6^ are expressed by neurons and cells of the immune system and gastrointestinal (GI) tract.^7,8^ Exogenous synthetic and semi-synthetic opioids, introduced orally, nasally, or intravenously, bind to these ORs with distinctive affinities. Exogenous opioids are grouped by their function: agonists, such as morphine, heroin, and fentanyl;^5,9^ rescue antagonists, that include naloxone and naltrexone,^10^ and the long-acting agonists, methadone, and buprenorphine used in medication-assisted treatment to prevent withdrawal symptoms and reduce cravings.^10–12^ As a result of the high opioid prescription rates in the 1990s, greater than 70% of heroin addictions originate from prescription opioids.^2^ Methadone, the pharmacological component of medication-assisted treatment, alleviates moderate to severe pain and treats chronic opioid misuse.^10,11^ The side effects of most opioids include dizziness, drowsiness, impaired cognition, and coordination, constipation, nausea, and vomiting.^11,12^ Gastrointestinal distress brought on by these latter symptoms is referred to as opioid-induced bowel dysfunction (OIBD). We hypothesize that OIBD is associated with changes in the intestinal microenvironment, including dysbiosis of the luminal microbiota, imbalance in the bacterial metabolite profile and damage to the intestinal epithelial barrier.

The intestinal microbiome plays a major role in gastrointestinal health and disease. It is essential for the degradation of food products,^13^ the production of bacterial metabolites,^14,15^ defense against pathogens,^16^ and maintenance of intestinal barrier integrity.^17,18^ Microbial homeostasis is attained with balanced levels of the core taxa, *Actinobacteria, Bacteroidetes, Firmicutes, Proteobacteria*, and *Verrucomicrobia*.^16,19^ Several factors including diet, lifestyle, alcohol and drug use, and immunodeficiency, can affect this homeostasis.^20,21^ Disruption and imbalance of bacterial homeostasis, known as dysbiosis,^7^ is linked to a variety of diseases, like inflammatory bowel disease (IBD), cardiovascular diseases, and obesity,^19,22^ and can lead to altered metabolic function, reduced diversity, and increased abundance of pathobionts.^23^ Fecal metabolites are small molecules secreted by the microbiota that modulate host metabolism at local and distant sites.^14,15^ Among the most studied are short-chain fatty acids (SCFAs), primarily acetate, propionate, and butyrate, involved in energy intake and expenditure,^24,25^ gut motility,^25^ maintenance of epithelial barrier function, and regulation of intestinal immunity.^26^

The intestinal barrier consists of an epithelial cell monolayer, where the paracellular space between adjacent epithelial cells is sealed by the tight junctional complex that selectively inhibits solute and water flow.^18,27^ On the apical surface of the epithelium the mucus layer protects the host from pathogenic organisms. The epithelium overlays the lamina propria, which contains a myriad of immune cells. Continuous opioid exposure in rodents leads to decreased gastrointestinal motility and transit, microbial dysbiosis, increased permeability, and microbial translocation.^28–30^ Our goal is to define the mechanisms that lead to the gastrointestinal disruption underlying OIBD. We have characterized the gut microbiome and bacterial metabolite profile of individuals in a methadone treatment program and subsequently examined how methadone and its resultant bacterial metabolites affect the intestinal epithelial barrier *in vitro*.

## Results

### Decreased fecal bacterial diversity and composition in methadone-treated individuals

**Figure 1.**
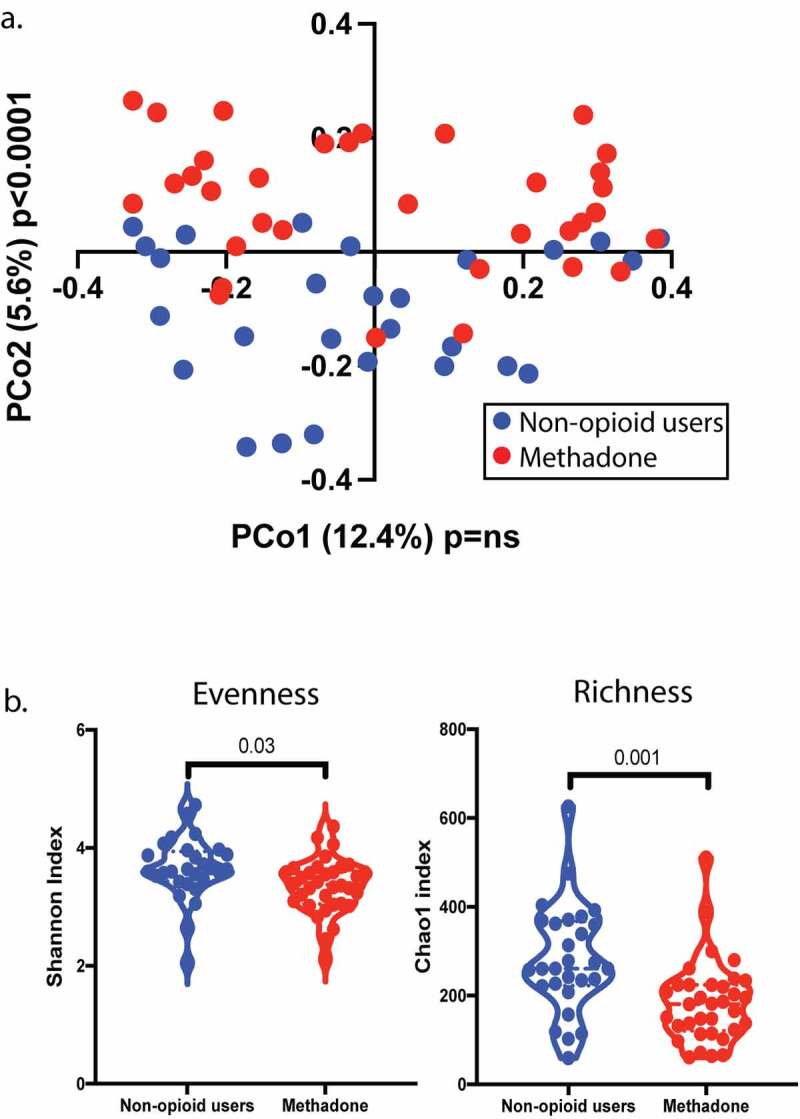
β- and α-diversity of the fecal microbiome is modulated by methadone treatment. a, Principal component analysis (PCA) revealed changes in the bacterial composition between the non-opioid users (blue) and methadone treated individuals (red) (PCo2 *p* < .0001). b, Bacterial biodiversity analysis showed decreased α-diversity in evenness (*p* = .03) and richness (*p* = .001) in the methadone cohort (red circles) using the Shannon and Chao1 index, respectively compared to non-opioid users (blue circles). Statistical significance was determined using a two-tailed unpaired t-test (a and b). A violin plot was used to represent the distribution of fecal samples from 28 non-opioid users and 34 methadone-treated individuals (dashed lined indicates the median, dotted line indicates quartiles)

To explore alterations in the gut microbial community structure during methadone treatment, we sequenced the V3-V4 region of the bacterial 16S rRNA from human fecal samples and performed taxonomic profiling between 62 demographically matched non-opioid users and methadone-treated individuals (Table 1, Supplemental Table S1). Unbiased analysis of β-diversity showed striking differences in microbial composition between the groups, especially in PCo2 (*p* < .0001) (Figure 1a). Dysbiosis was confirmed upon analysis of two additional measurements of biodiversity: evenness (Shannon, *p* = .03) and richness (Chao1, *p* = .001) (Figure 1b). Decreased diversity for both indices was observed in samples from methadone-treated individuals compared to non-opioid users. These results indicate that methadone treatment associates with lower diversity of bacterial communities in the GI tract.

**Table 1.** Study population demographics

Demographics	Non-opioid users	Methadone	p-value
Donors (Female/Male/Not specified)	28 (11/13/4)	34 (15/16/3)	ns^#^
Average age (range)	40 (26–62)	46 (32–58)	ns*
**Race/Ethnicity**			
Caucasian	16	22	ns^#^
Black/African American	0	2	
Asian	2	0	
Hispanic/Latino	5	4	
Other	0	3	
Prefer not to respond	1	0	
Not specified	4	3	

Statistical Test performed: *t-test, #Chi-square

### Chronic opioid use disrupts the fecal microbiota

**Figure 2.**
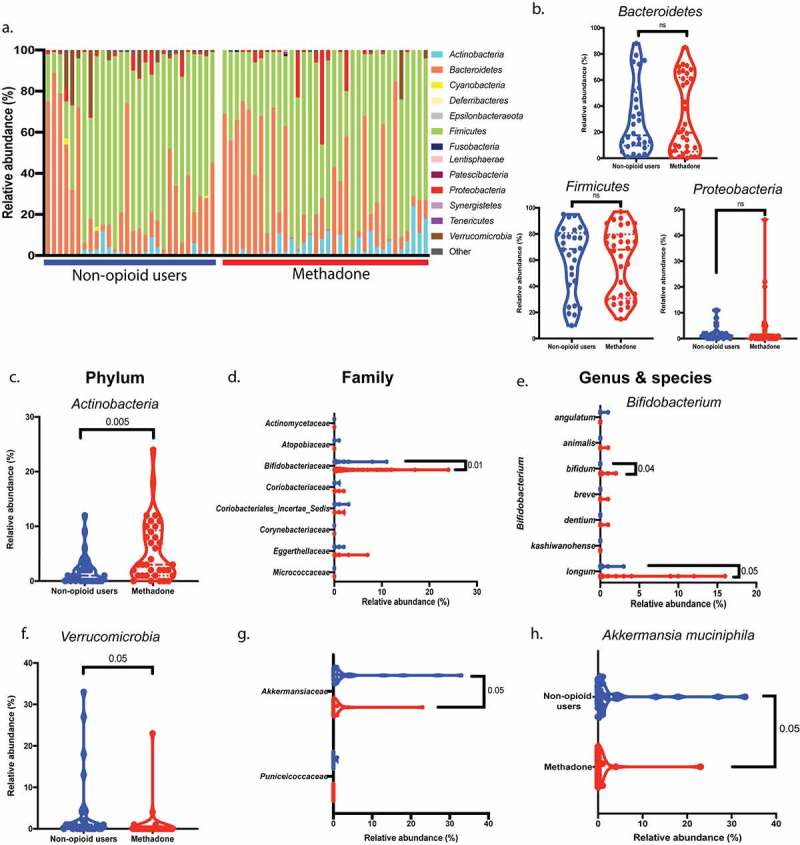
Increased fecal *Actinobacteria* and decreased *Verrucomicrobia* abundance with methadone treatment. a, Analysis of bacterial 16S rRNA from fecal samples revealed 13 phyla from the methadone-treated cohort (horizontal red bar) and non-opioid users (horizontal blue bar), in which each column represents an individual donor and the top 8 most abundant bacteria phyla. b, c, f, The relative abundance of the core microbiota, *Bacteroidetes, Firmicutes, Proteobacteria, Actinobacteria*, and *Verrucomicrobia* was compared between groups. *Bacteroidetes, Firmicutes*, and *Proteobacteria* (b) from methadone-treated individuals (red circles) showed no statistical difference in relative abundance compared to non-opioid users (blue circles). *Actinobacteria* was significantly increased (c) (*p* = .005) and *Verrucomicrobia* significantly decreased (f) (*p* = .05) in the methadone group (red circles). d, Within the *Actinobacteria* phylum, eight bacterial families were identified. Only *Bifidobacteriaceae* (*p* = .01) showed increased relative abundance in the methadone-treated group (red circles). e, *Bifidobacterium bifidum* (*p* = .04) & *Bifidobacterium longum* (*p* = .05) were increased in the methadone-treated group (red circles). g,h, Within the *Verrucomicrobia* phylum, *Akkermansiaceae* family (*p* = .05) (g) and the *Akkermansia muciniphila* species (*p* = .05) (h) were decreased in methadone-treated individuals (red circles) compared to non-opioid users (blue circles). Statistical significance was determined using a two-tailed unpaired t-test (b-h). Violin plots were used to represent the distribution of fecal samples from 28 non-opioid users (blue) and 34 methadone-treated individuals (red) for the taxonomic classifications (phylum, family, genus, and species) (dashed lined indicates the median, dotted line indicates quartiles)

To characterize the dysbiosis of methadone-treated individuals, we identified bacterial communities associated with decreased bacterial diversity. The gut microbes detected in this study consisted of 13 bacterial phyla (Figure 2a) predominantly *Actinobacteria, Bacteroidetes, Firmicutes, Proteobacteria*, and *Verrucomicrobia*. While abundance of certain bacterial phyla is similar between the two groups (Figure 2b), two phyla of the core microbiota were significantly altered by chronic methadone exposure *in vivo*, namely *Actinobacteria* and *Verrucomicrobia*. A significant increase in the relative abundance of *Actinobacteria* (*p* = .005, Figure 2c) was seen in methadone-treated individuals. Eight bacterial families within this phylum were identified (Figure 2d), with fecal *Bifidobacteriaceae* significantly increased (*p* = .01) in methadone-treated individuals compared to non-opioid users. Within this bacterial family, the species *Bifidobacterium bifidum* and *Bifidobacterium longum* were significantly elevated in methadone-treated individuals (Figure 2e). Strikingly, a subtle yet significant decrease in relative abundance of the *Verrucomicrobia* phylum (*p* = .05, Figure 2f) was observed in samples from methadone-treated individuals. Within this phylum, *Akkermasiaceae* (*p* = .05, Figure 2g) was identified as the bacterial family decreased in methadone-treated individuals. From this bacterial family, the *Akkermansia muciniphila* species (Figure 2h) was found to be decreased in the methadone-treated group (*p* = .05).

We also evaluated whether these differences in bacterial communities were associated with the duration of methadone treatment (short – ≤5 years *vs*. long – ≥10 years; Supplemental Figure S1). A significant increase in the relative abundance of *Bacteroidetes* (*p* < .0001) was observed in individuals after long-term methadone treatment compared to that of their short-term counterparts. Conversely, the relative abundance of *Firmicutes* (*p* = .0002) was significantly decreased in individuals with long duration of methadone treatment. *Actinobacteria* (*p* = .07), *Proteobacteria* (*p* = .24), and *Verrucomicrobia* (*p* > .99) showed no statistical variation due to the duration of methadone treatment. Overall, duration of methadone treatment is an important contributing factor in the composition of the fecal microbiota after opioid use.

### Metabolomic profile is shifted with methadone use

**Figure 3.**
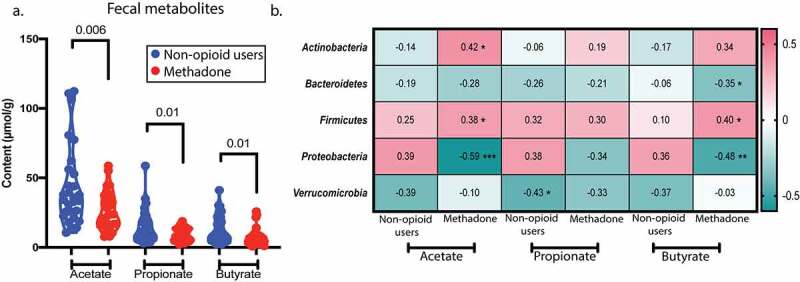
Fecal SCFA profile is altered with methadone use and correlates with core microbiota. a, SCFA content in feces was assessed by gas chromatography-mass spectrometry. Acetate (*p* = .006), propionate (*p* = .01), and butyrate (*p* = .01) levels are decreased in the methadone cohort (red circles). b, Spearman correlation between fecal SCFAs and the relative abundance of the core microbiome from non-opioid users and methadone-treated individuals. Heatmap shows Spearman r coefficient values, and asterisks denotate statistical significance. Acetate correlates positively with *Actinobacteria* (*p* = .02) and *Firmicutes* (*p* = .03) and negatively with *Proteobacteria* (*p* = .0004) in methadone-treated individuals. Butyrate correlates positively with *Firmicutes* (*p* = .03) and negatively with *Bacteroidetes* (*p* = .05) and *Proteobacteria* (*p* = .006) in methadone-treated individuals. p ≤ 0.05 (*), p ≤ 0.005 (**), p ≤ 0.0005 (***). Statistical significance was determined using a two-tailed unpaired t-test (a). A violin plot was used to represent the distribution of fecal samples from 24 non-opioid users and 32 methadone-treated individuals (dashed lined indicates the median, dotted line indicates quartiles)

In addition to alterations in the bacterial communities, changes in metabolomic profile are associated with methadone use. The content of bacterial metabolites, specifically SCFAs, was measured using gas chromatography-mass spectrometry (GC-MS) from human feces (Figure 3a) and plasma (Supplemental Figure S2a). The feces of methadone-treated individuals showed a significant decrease in acetate (*p* = .006), propionate (*p* = .01), and butyrate (*p* = .01) when compared to that of non-opioid users (Figure 3a), while plasma propionate (*p* = .05) levels were increased (Supplemental Figure 2a).

Spearman correlations reveal relationships between fecal SCFA content and bacterial communities (Figure 3b). *Actinobacteria* (*p* = .02) and *Firmicutes* (*p* = .03) positively correlate with fecal acetate in methadone-treated individuals, while *Proteobacteria* (*p* = .0004) negatively correlates with acetate. As seen with acetate, butyrate positively correlates with *Firmicutes* (*p* = .03) and negatively correlates with both *Bacteroidetes* (*p* = .05) and *Proteobacteria* (*p* = .006) in methadone-treated individuals. Similarly, when analyzing metabolites in plasma (Supplemental Figure S2b), *Firmicutes* positively correlate with acetate (*p* = .03) and butyrate (*p* = .04) and *Bacteroidetes* negatively correlate with acetate (*p* = .008) in methadone-treated individuals. Overall, these results indicate that the compromised fecal microbiota and the altered metabolite profile are interconnected with chronic opioid exposure, but not dependent on duration of treatment (data not shown). In particular, the increased relative abundance of *Actinobacteria* and its positive correlation with fecal and blood concentrations of all three SCFAs in the methadone group (pink) contrasts with its negative correlation in non-opioid users (green). Additionally, *Firmicutes* and *Bacteroidetes* cooperate to modulate acetate and butyrate content in feces and blood during methadone exposure *in vivo.*

**Figure 4.**
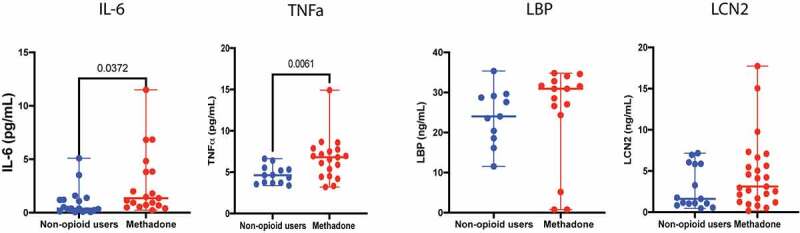
Methadone treatment associates with increased plasma IL-6 and TNF-α. Levels of plasma IL-6 (*p* = .03), TNF-α (*p* = .006), LBP (*p* = ns), and as well as fecal LCN2 (*p* = ns) between non-opioid users (blue) and methadone-treated individuals (red) were measured. Statistical significance was determined using a two-tailed unpaired t-test

Plasma levels of specific immune mediators, including interleukin-6 (IL-6), C-reactive protein (CRP), Lipopolysaccharide binding protein (LBP), and intestinal-type fatty acid-binding protein (IFABP), are increased in HIV+ and IBD patients experiencing dysbiosis and intestinal permeability.^31^ We therefore examined plasma concentrations of these markers as well as other important inflammatory mediators that included IL-8, IL-1β, MIP1α, TNFα, and fecal Lipocalin 2 (LCN2) in the methadone-treated population compared to non-opioid users (Figure 4, Supplemental Table S2) to identify potential biomarkers associated with chronic opioid use. IL-6 (*p* = .03) and TNFα (*p* = .01) were significantly increased in the methadone cohort, while CRP (*p* = .26), LBP (*p* = .83), IFABP (*p* = .64), MIP1α (*p* = .36), IL-1β (*p* = .96), IL-8 (*p* = .41), and LCN2 (*p* = .19) revealed no differences between groups. Spearman analysis between these immune mediators and fecal bacterial abundance (Supplemental Figure S2c), fecal SCFAs (Supplemental Figure S2d), and plasma SCFAs (Supplemental Figure S2e) in the methadone cohort revealed significant correlations. MIP1α negatively correlated with plasma acetate (*p* = .01) and butyrate (*p* = .01), and lipocalin 2 negatively correlated with *Bacteroidetes* (*p* = .04) and *Verrucomicrobia* (*p* = .04) in methadone-treated individuals. Some correlations among these parameters were observed in the non-opioid users, but their importance to opioid use is unclear. In non-opioid users plasma IL-6 and CRP positively correlated with *Firmicutes* (*p* = .04, *p* = .01, respectively), fecal acetate, propionate, and butyrate, and CRP negatively correlated with *Bacteroidetes* (*p* = .01). LBP negatively correlated with *Actinobacteria* (*p* = .04), and TNFα (*p* = .01) and I-FABP (*p* = .03) negatively correlated with *Verrucomicrobia*, while I-FABP correlated positively with plasma butyrate (*p* = .03) in non-opioid users. These results suggest that circulating levels of immune mediators in healthy adults may vary depending on the abundance of various bacteria and their SCFA metabolites, and that reduction in microbial diversity due to methadone use may disrupt the balance between the gut microbiota and key modulators of the immune system during chronic opioid use.

### Akkermansia muciniphila *spent media components, but not short-chain fatty acids (SCFAs) nor outer membrane vesicles (OMVs), modulate intestinal barrier integrity*

**Figure 5.**
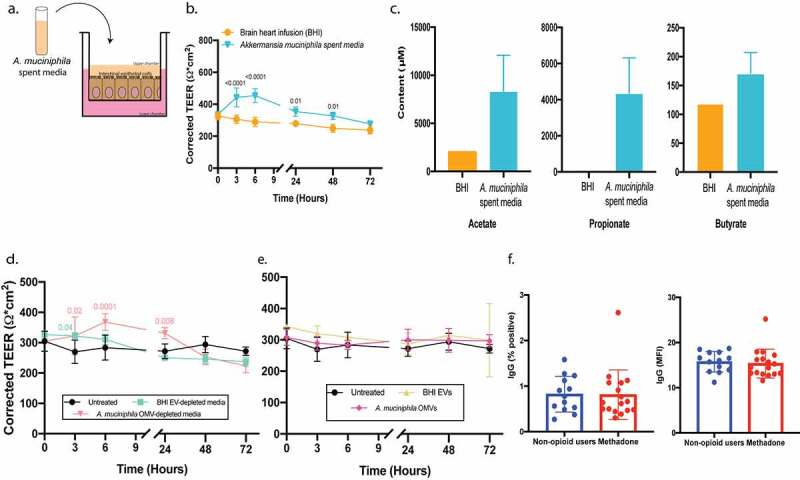
Components of *Akkermansia muciniphila* spent media, not short-chain fatty acids (SCFAs) or outer membrane vesicles (OMVs), modulate intestinal barrier integrity. a, TEER of an intestinal epithelial Caco-2 monolayer in a transwell culture was measured after exposure to *Akkermansia muciniphila* spent media as noted in the model diagram. b, *Akkermansia muciniphila* spent media (blue) added to the upper chamber of the transwell increased epithelial barrier integrity at 3 and 6 h (*p* < .0001) compared to control BHI (orange). c, Short-chain fatty acid content in *Akkermansia muciniphila* spent media (n = 2). d, *Akkermansia muciniphila* OMV-depleted media (light pink) added to the upper chamber of the transwell increased epithelial barrier integrity at 3 (*p* = .02) and 6 h (*p* < .0001) compared to untreated cells (black) and cells exposed to EV-depleted BHI media (light blue). e, *Akkermansia muciniphila* OMVs (dark pink) and BHI EVs (light yellow) added to the upper chamber of the transwell had no effect on barrier integrity compared to untreated cells (black). f, Percent positive (left) and MFI (right) levels of IgG against *Akkermansia muciniphila* in non-opioid users and methadone-treated individuals was measured using flow cytometry. Statistical significance was evaluated with a two-way ANOVA with Sidak correction (b), Tukey correction (d & e), and Welch’s t-test (f). Unless otherwise noted, mean and standard deviation from n = 3 biological replicates are shown

Given the decreased relative abundance of *Akkermansia muciniphila* and SCFAs with methadone treatment, we studied the ability of this bacterium to modulate intestinal barrier integrity. It is difficult to co-culture an intestinal epithelial monolayer under the anaerobic conditions required by *A. muciniphila*. We instead cultured the bacterium *in vitro* anaerobically for four days and harvested the spent media. To characterize the modulating effects of *A. muciniphila* spent media on barrier integrity, we established an *in vitro* cell culture model using an intestinal epithelial monolayer of Caco-2 BBe cells grown in a transwell configuration.^32^ *A. muciniphila* spent media added to the apical surface of the epithelial monolayer (Figure 5a), replicating the morphology of the gut microenvironment, increased intestinal barrier integrity at 3 and 6 h (*p* < .0001) (Figure 5b) compared to control medium.

To identify the key modulators in *A. muciniphila* spent media strengthening the intestinal barrier, we investigated its metabolite content. The concentration of the SCFAs acetate and propionate are higher in *A. muciniphila* spent media compared to control medium (Figure 5c). Therefore, we tested the ability of SCFAs to modulate barrier integrity. The intestinal epithelial monolayer was treated with similar concentrations of acetate, propionate, or butyrate found in spent media (Supplemental Figure 3a). Only propionate showed a minimal effect on epithelial barrier integrity at 24 h (*p* = .04, Supplemental Figure S3a). These findings indicate that SCFAs do not directly modulate barrier integrity, and more importantly, the kinetics of the minimal effect of propionate are different than those observed with *A. muciniphila* spent media.

Several studies have indicated that *Akkermansia muciniphila* outer membrane vesicles (OMVs) have the capacity to modulate tight junction proteins.^17,33^ Therefore, *A. muciniphila* OMVs were isolated, purified, and concentrated from the spent media using tangential flow filtration (TFF) (Supplemental Figure S3b) and quantified with microfluidic resistive pulse sensing (MRPS) (Supplemental Figure S3c). As expected after TFF, the concentration of mammalian-derived extracellular vesicles (EVs) and *A. muciniphila*-derived OMVs was decreased in the depleted media and adjusted to be similar to that of the input media (Supplemental Figure S3c). OMV-depleted media or purified OMVs were added to the apical surface of an intestinal epithelial cell monolayer to evaluate their role in modulating barrier integrity. *A. muciniphila* OMV-depleted media increased intestinal barrier integrity at 3 (*p* = .02), 6 (*p* = .0001), and 24 (*p* = .008) h with the same kinetics and potency as spent media (Figure 5d, compare to Figure 5b), while *A. muciniphila* OMVs had no effect on barrier integrity (Figure 5e). This indicates that the effects are likely due to metabolites not associated with OMVs.

To evaluate a role for intestinal barrier integrity in the communication between the gut microbiota and the systemic immune response, IgG levels directed against *Akkermansia muciniphila* were assessed by flow cytometry. Similar levels of anti-*A. muciniphila* IgG (percent positive cells and mean fluorescence intensity (MFI); *p* = .9, .7, respectively,) were observed between non-opioid users and methadone-treated individuals (Figure 5f). These results are in line with our observation of increased barrier integrity with *Akkermansia muciniphila* spent media, indicating that this organism may limit its own microbial translocation across the epithelium and further interaction with the immune system.

### Akkermansia muciniphila *spent media and methadone independently modulate intestinal barrier integrity*

**Figure 6.**
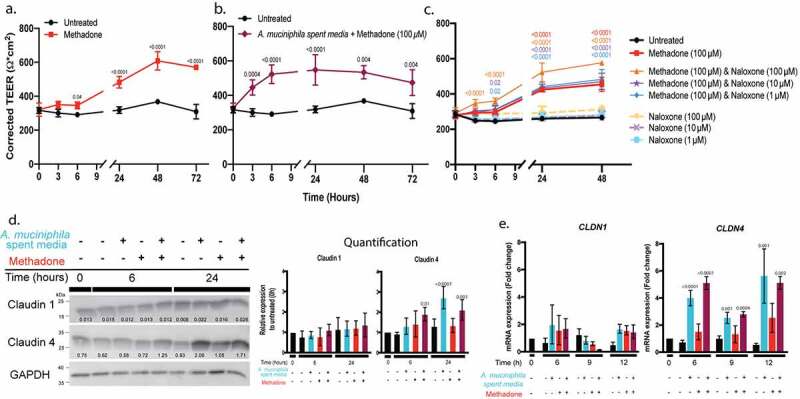
*Akkermansia muciniphila* spent media and methadone increase intestinal epithelial barrier integrity *in vitro* independently with distinct kinetics. a, 100 µM methadone (red) added to the lower chamber of the transwell increased epithelial barrier integrity at 24, 48, and 72 h (*p* < .0001) compared to an untreated Caco-2 monolayer (black). b, The combination of *Akkermansia muciniphila* spent media and 100 µM methadone (maroon) added to the upper and lower chambers, respectively, increased barrier integrity with distinct kinetics: 3 h (*p* = .0004) and 6 h (*p* < .0001) due to *Akkermansia muciniphila* spent media and 24 h (*p* < .0001), 48 h (0.004), and 72 h (*p* = .004) in response to methadone. c, TEER of the Caco-2 monolayer was measured in the presence of graded concentrations of the OR rescue antagonist naloxone alone or in combination with 100 µM methadone. High dose naloxone enhanced the methadone-stimulated increase in barrier integrity (*p* < .0001), when compared to an untreated Caco-2 monolayer, naloxone alone, or methadone alone. d, Protein expression and quantification for claudin 1 and claudin 4 after a Caco-2 monolayer was treated with Akkermansia *muciniphila* spent media (blue), methadone (red), or the combination (maroon) for 0, 6, and 24 h. Densitometric quantification normalized to GAPDH, relative to the untreated sample (black). e, A Caco-2 monolayer was stimulated with *A. muciniphila* spent media (blue), 100 µM methadone (red), or the combination (maroon) for 6, 9, and 12 h. mRNA expression (fold change measured by RT-qPCR) is relative to untreated sample (black), set to 1. Statistical significance was determined using a two-way ANOVA with Sidak correction (a, b), Tukey correction (c), and one-way ANOVA with Dunnett correction (d-e). Mean and standard deviation from n = 3 biological replicates (a-c, e) and n = 5 biological replicates (d) are shown

Due to the decreased levels *of Akkermansia muciniphila* in methadone-treated individuals, we also explored the direct effects of methadone on barrier integrity. The monolayer was exposed to 1, 10, or 100 µM methadone at the basolateral surface – the equivalent of supplying methadone through the blood stream. In a dose dependent manner (Supplemental Figure S4) 100 µM methadone increased the strength of the intestinal barrier at 24, 48, and 72 h (*p* < .0001) (Figure 6a). In addition to methadone, we explored the effect of other exogenous opioids that selectively target the µ-OR on barrier integrity. Neither morphine nor [D-Ala,^2^ N-Me-Phe,^4^ Gly^5^-ol]-Enkephalin acetate salt (DAMGO) modulated barrier integrity (data not shown). Similarly, the κ-OR selective agonist BRL52537 hydrochloride and the δ-OR selective agonist [D-Pen 2,5] Enkephalin (DPDPE) did not regulate transepithelial resistance. The distinct kinetics of *A. muciniphila* spent media (Figure 5b) and methadone (Figure 6a) suggest different molecular mechanisms. Nonetheless, we explored if the combined effects of *A. muciniphila* spent media and methadone on the barrier might be additive or cooperative (Figure 6b). The combination treatment increased epithelial barrier integrity at 3 (*p* = .0004), 6 (*p* < .0001), 24 (*p* < .0001), 48 (*p* = .004), and 72 (*p* = .004) h, suggesting that the components of *A. muciniphila* spent media and methadone modulate intestinal barrier integrity independently with different kinetics.

To determine if the effect of methadone on epithelial barrier integrity is receptor-mediated, we blocked methadone binding to opioid receptors with the rescue antagonist naloxone (Figure 6c). Unexpectedly, fixed concentrations of methadone exposure to the monolayer in the presence of graded concentrations of naloxone showed a dose dependent increase in intestinal barrier integrity at 6 (*p* = .02, <0.0001), 24, and 48 (*p* < .0001) h, greater than methadone alone, when compared to untreated cells and individual concentrations of naloxone (Figure 6c; Supplemental Table S3). Naloxone alone had no effect on epithelial permeability. These surprising observations indicate a complex interaction between methadone and its receptors on intestinal epithelial cells that requires further characterization beyond the scope of this study.

To highlight a potential mechanism by which *A. muciniphila* spent media or methadone increases intestinal barrier integrity, we examined the tight junction (TJ) complex, which regulates paracellular permeability. Transepithelial electrical resistance (TEER) is a measure of the TJ pore pathway, regulated by expression of the integral membrane proteins claudin 1 and claudin 4, which form extracellular loops to create the TJ pore. We followed the expression of claudin 1 and claudin 4 proteins by immunoblot in epithelial monolayers treated with *A. muciniphila* spent media or methadone or the combination over a 24 h period, the time frame within which each agonist stimulates an increase in TEER. No treatment affected claudin 1 protein expression. Claudin 4 protein expression was significantly increased by *A. muciniphila* spent media alone at 24 h and by *A. muciniphila* spent media with methadone at 6 and 24 h (Figure 6d). *A. muciniphila* spent media increases TEER in the monolayer at 3 and 6 h, so the increase in claudin 4 protein expression is unlikely to be related to the rapid change in barrier integrity, suggesting two levels of regulation. The elevated expression of claudin 4 protein was confirmed by demonstrating that *CLDN4*, but not *CLDN1*, gene expression is stimulated at 6, 9, and 12 h (Figure 6e). The combination of *A. muciniphila* spent media and methadone stimulates *CLDN4* mRNA expression to the same degree and with the same kinetics as *A. muciniphila* spent media alone, confirming the findings presented in (Figure 6b), that each agonist is acting independently.

## Discussion

Over the past several decades the United States has experienced a devastating opioid epidemic.^2^ Efforts to address this crisis include improved management of prescription opioids, development of non-addictive analgesics, and expansion of medication-assisted treatment programs. Participants in these programs, and most opioid users, experience a vast array of symptoms including gastrointestinal motility disorders^7,34,35^ due to OIBD, a debilitating side of effect of chronic opioid use.^4,6,34,35^ This study focused on the impact of chronic methadone use on the gut microbiome, the consequent bacterial metabolite profile, and intestinal barrier integrity.

Human studies investigating the effects of chronic opioid use on the gut microbiome are limited^36–40^ with conflicting results, as some report an increase in microbial diversity,^39,40^ while others showed no significant changes in α diversity,^37^ yet changes in microbial composition are consistent across these studies.^36–39^ Principal coordinate analysis of our results reveals distinct differences in the microbiome of methadone-treated individuals, particularly in the second dimension. These differences are further highlighted by a decrease in evenness and richness in the α diversity of this cohort. As with any population study, it is likely that other unmeasured socioeconomic factors may also distinguish these two cohorts, although we tried to minimize such differences by matching the participants according to age, sex, race, antibiotic use, diarrhea, constipation, viral infection, and lack of systemic inflammation.

In line with diversity analyses, we observed a significant increase in the *Bifidobacterium* genus (*Actinobacteria* phylum) in methadone-treated individuals, which is consistent with the existing opioid literature.^36–39^ *Bifidobacterium*, a highly represented genera in the adult microbiota,^41–45^ is known to produce the SCFA acetate.^45^ Interestingly, we observed a decrease in the content of fecal acetate and a positive correlation between *Actinobacteria* and fecal acetate. Mouse studies show that a reduction in SCFAs enhances colonic fluid and sodium absorption, thereby aggravating constipation. Additional studies show the beneficial effect of *Bifidobacterium* as a probiotic to alleviate constipation.^41–45^

We report that *Akkermansia muciniphila* (*Verrucomicrobia* phylum) was significantly decreased in methadone-treated individuals. *A. muciniphila* is a key modulator of host health by triggering host metabolic and immune responses^46,47^ as well as beneficial microbial networks.^46,47^ This species is found in the healthy gut (1–3%),^48,49^ and its depletion is associated with IBD, appendicitis, obesity, diabetes, among other disorders.^13,47,49^ *A. muciniphila*, proposed as a next-generation probiotic,^46^ is characterized as a mucin degrader because it uses colonic mucin as an energy source.^22^ *A. muciniphila* also produces SCFAs and strengthens intestinal epithelial integrity^17,49,50^ by adhering to enterocytes in different stages of differentiation. Collectively, depletion of *A. muciniphila* in the methadone-treated gut might lead to a loss of mucin degradation, possibly supporting the expansion of pathobionts disrupting the intestinal barrier, thus contributing to OIBD.

Reports that *A. muciniphila* strengthens barrier integrity *in vitro*^49^ stimulated our interest in defining its mechanism of action. Characterizing the effects of *A. muciniphila* in mammalian cells is limited by its restricted oxygen tolerance, preventing aerobic co-cultures.^13,47,50^ For this reason, our study focused on metabolites released by the bacteria after anaerobic growth. As with many other commensal organisms *A. muciniphila* produces SCFAs, which regulate cell proliferation, gut motility, and maintenance of gut barrier function.^24,25^ We observed a positive correlation between fecal SCFAs and plasma levels of IL-6 and CRP, two markers of inflammation, in non-opioid using adults, which is not seen in the methadone cohort. While fecal levels of propionate are decreased in methadone-treated individuals, plasma levels of propionate are increased, suggesting a selective transport of this SCFA through the methadone-compromised epithelium. When we evaluated the ability of SCFAs to directly modulate intestinal barrier integrity, only propionate stimulated a minimal strengthening of the pore pathway, suggesting that SCFAs may more effectively control gut barrier function indirectly through their immunomodulatory and HDAC inhibitory activities.^51,52^

We broadened our approach by investigating the total metabolic profile of *A. muciniphila*, evaluating the effects of unfractionated spent media on barrier integrity. Our results show that *A. muciniphila* spent media increases barrier resistance to ion flux within 3 to 6 h, thereby strengthening the pore pathway established by the TJ complex. To investigate *A. muciniphila* regulation of the TJ complex directly, we demonstrated that spent media stimulates claudin 4 mRNA and protein expression at 6–12 and 24 h, respectively, far later than its induction of the barrier’s electrical resistance at 3–6 h. These findings indicate that *A. muciniphila* regulates the intestinal barrier by two distinct mechanisms. These results are consistent with literature that reports *A. muciniphila* metabolites regulate genes and transcription factors involved in cellular metabolism.^13,46^ In addition, *A. muciniphila* OMVs were reported to influence gut permeability by regulating tight junction proteins.^17,33^ OMVs exhibit diverse roles in transferring of genetic material and proteins to the host.^17^ However, our findings do not provide evidence for a role of OMVs, as purified OMVs from *A. muciniphila* spent media did not affect barrier integrity, and OMV-depleted spent media was equally effective as the unfractionated spent media at inducing increased paracellular resistance. *A. muciniphila* secretes a variety of fermentation products, including essential amino acids, polyphenols, and various unidentified metabolites, in addition to SCFAs.^22^ Of particular interest is the bacterial outer membrane protein Amuc_1100, which in mouse studies protects the integrity of the epithelium via TLR2^22^ enhancing transepithelial resistance.^50^ We hypothesize that *A. muciniphila* metabolites (i) modulate the pore pathway at 3–6 h, (ii) stimulate tight junction mRNA (6–12 h) and protein expression (24 h), and (iii) include the production of SCFAs. In the future, these observations will be examined using human Air-Liquid Interface (ALI) organoid models that offer a variety of intestinal cell subtypes that recapitulate *in vivo* counterparts.^53–55^

The direct effect of methadone on modulating intestinal barrier integrity *in vitro* has not been studied. From our results, methadone strengthened the intestinal barrier >24 h after exposure. In contrast, other exogenous opioids, such as morphine and [D-Ala,^2^ N-Me-Phe,^4^ Gly^5^-ol]-Enkephalin acetate salt (DAMGO) that selectively target the μ-OR did not modulate barrier integrity (data not shown). A similar lack of effect was observed for the κ-OR selective agonist BRL52537 hydrochloride and the δ-OR selective agonist [D-Pen 2,5] Enkephalin (DPDPE). The kinetics of methadone’s effect on the barrier are quite distinct from those of *A. muciniphila* spent media. Its activity is neither additive nor cooperative with *A. muciniphila* spent media, and methadone does not stimulate claudin 1 or 4 mRNA or protein expression. Taken together we propose that methadone’s contribution to barrier integrity is via a mechanism distinct from *A. muciniphila*. Surprisingly, the OR antagonist naloxone did not inhibit methadone activity. Instead, the combination of naloxone and methadone was more potent in strengthening the intestinal barrier than methadone alone, suggesting that naloxone might be acting as an inverse agonist.^56^

Two limitations of this study are that it lacks (i) longitudinal kinetics and (ii) fecal microbiota samples before opioid use, both of which present major sociological challenges in light of the population being studied. Thus, a causative role of methadone on the gut microbiome and bacterial metabolite profile cannot be drawn. Nonetheless, we present a strong association between chronic opioid use and dysbiosis of the gut microenvironment. In the future, we will expand these studies to include participants pre- and post-initiation of opioids to eliminate any bias in the observed effect due to continuous exposure to opioids.

In conclusion, we report that the impact of methadone treatment on the gut microbiome associates with an imbalance in key bacterial communities required for production of SCFAs, degradation of mucus, and for maintenance of barrier integrity. A decrease in fecal SCFAs in methadone-treated individuals is associated with altered plasma levels of immune mediators, whose expression is regulated by the HDAC inhibitory activity of SCFAs. We propose that components in *A. muciniphila* spent media modulate intestinal barrier integrity *in vitro* by both directly strengthening the pore pathway and regulating tight junction gene expression. This study provides essential information regarding the therapeutic potential of *A. muciniphila* and warrants development of new clinical strategies that target normalizing the gut microbiome of individuals affected by chronic opioid use.

## Methods

### Study subjects

A total of 62 adults, 28 non-opioid users and 34 methadone-treated individuals, were recruited from the Cleveland Treatment Center and Case Western Reserve University. The study was approved by the Internal Review Board at University Hospitals Cleveland Medical Center (Protocol # 08–11-05) and written informed consent was obtained from each participant. Samples and self-reporting documents were de-identified before use. Volunteers were matched for age, sex, race, and ethnicity (Table 1) and self-reported length of methadone treatment, viral infections (HIV, HCV, and HBV), other medications, and type of bowel movements: diarrhea or constipation (Supplemental Table S1).

### Sample collection and processing

Peripheral blood was collected in BD Vacutainer® (Fisher Scientific, 02–689-6) tubes containing heparin, and plasma isolated by removing cells by centrifugation at RT for 10 min and stored at −20°C. Stool samples were collected in a dry container (ThermoFisher Scientific, 22–010-1159) and stored at −20°C.

DNA was extracted from stool using the QIAamp Fast DNA Stool Mini Kit (Qiagen, 51604) as described by the manufacturer. DNA was eluted from the silica-based membrane with 150 µL of ATE Buffer (Qiagen, 51604) and stored at −20°C.

### 16S rRNA library preparation and quantification

Bacterial DNA was quantified using the Qubit Broad Range assay (ThermoFisher Scientific, Q32853) and 12.5 ngs (5 ng/µl) were used for library preparation following the Illumina MiSeq *16S Metagenomic Sequencing Library Preparation* protocol (targeting the V3 and V4 region of the 16S rRNA gene).^19^ The DNA library was quantified and normalized to 4 nM before pooling, and 5% PhiX (Illumina, FC-110-3001) was added as an internal control. The library was sequenced using the MiSeq Reagent Kit v3 2 × 300 cycles (Illumina, MS-102-3003). Fastq files were generated for further analysis.

### Quality control and pipeline analysis

Fastq files obtained through BaseSpace (Illumina) were analyzed using Nephele^57^ and Dada2.^58,59^ Low-quality sequences were removed and primers truncated. Sequences were clustered to collapse similar sequences (with at least 99% identity) to obtain Operational Taxonomic Units (OTUs). Silva v132 was used as the reference database. The resulting microbial abundance was used to perform additional analyses, such as α and β diversity and microbial composition. Associated data can be found at DOI: 10.17632/ch6rndwt5h.1.

### Targeted metabolomics – short-chain fatty acids

SCFAs from bacterial spent media, plasma, and fecal samples were measured by stable isotype dilution GC-MS/MS.^60^ Bacterial spent media (30 µL) or plasma (30 µL) were placed in a 200 µL GC vial with insert with 50 µL of 0.005 M NaOH. Twenty to forty mgs of feces were transferred to 2 mL cryovials and hydrated with 0.5 mL of 0.005 M NaOH. After vortexing and centrifugation, the supernatant was mixed with 50 µL of 2-Butanol/Pyridine (3:2) containing labeled internal standards. The carboxylic acids were derivatized with isobutyl chloroformate. After derivatization, the sample was mixed with hexane, and the hexane layer recovered for GC-MS analysis. Quantification of acetic, butyric, isovaleric, lactic, propionic, and succinic acids was performed on a TSQ-Evo triple quadrupole in tandem with the Trace 1310 gas chromatograph (Thermo Fisher Scientific) using MRM mode with the parent to daughter ion transitions listed in Supplemental Table S4.

### Immune mediators

Plasma IL-6, TNFα, IL-1β, and MIP1α (neat) (R&D Systems, HS600C, DTA00D, DLB50, DMA00), IL-8 (neat) (Abcam, ab46032), and CRP (1:100) (R&D Systems, DCRP00) were quantified following manufacturer’s instructions. LBP (1:100) (R&D Systems, DY870-05) and iFABP (1:10) (R&D Systems, DY3078) ELISA kits were supplemented with the DuoSet Ancillary Reagent Kit (R&D Systems, DY008). Fecal LCN2 was quantified following manufacturer’s instructions (Eagle Bioscience, NGL35-K01).

### *SFCAs and methadone for* in vitro *cell culture*

Acetate (Sigma Aldrich, S5636), propionate (Sigma Aldrich, P1880), butyrate (Sigma Aldrich, B5887), and methadone (Sigma Aldrich, M0267) were reconstituted in ultrapure distilled water, sterilized through a 0.2 μm filtered syringe, and serially diluted in EMEM containing 10% FBS (Fisher Scientific, MT35016CV).

### Bacterial strain and growth conditions

*Akkermansia muciniphila* (BAA-835), obtained from Dr. Hazen’s lab at the Cleveland Clinic, OH, was grown anaerobically at 37°C for 4 d in Brain Heart Infusion (BHI; Anaerobe systems, AS-872) under an atmosphere of 5% H_2_, 10% CO_2_, and 85% N_2_. Optical density (OD) (600 nm) was measured daily to follow the growth rate. Bacterial spent media, stored at −80°C, was separated from the cells using a 0.2 µm filter.

### Epithelial monolayer and transepithelial electrical resistance

The human colonic epithelial cell subclone, Caco-2 BBe, obtained from Dr. Jerrold Turner at Harvard University, Boston, MA, was grown in EMEM plus 10% FBS at 37°C in humidified 5% CO_2_. 60,000 cells were plated on transwell membranes and maintained at 37°C for 2 weeks to form an intact monolayer, with media changes every 2 days. Baseline TEER reading was recorded before any treatment and measured at 3, 6, 24, 48, or 72 h, as noted.

Graded concentrations of SCFAs, acetate, propionate, butyrate, in 100 µL EMEM plus 10% FBS, 100 µL of *Akkermansia muciniphila* spent media, or 100 µL sterile BHI were added to the upper chamber of the transwells. 700 µl of EMEM plus 10% FBS media was added to the lower chamber.

Graded concentrations of methadone, morphine, DAMGO, BRL52537, DPDPE, and naloxone (Sigma Aldrich, N7758) in 700 µl of EMEM plus 10% FBS media were added to the lower chamber, and 100 µL of EMEM plus 10% FBS media was added to the upper chamber of the transwells. For the combination treatment, 100 µL of *Akkermansia muciniphila* spent media was added on the upper chamber, while 100 µM methadone was added to the lower chamber.

### Tangential flow filtration and particle quantification

*Akkermansia muciniphila* OMVs were isolated, concentrated, and purified from 10 mL of spent media by tangential flow filtration with a KrosFlo KR2i TFF peristaltic pump system (Repligen, Waltham, MA), using a microKros polysulfone 500 kDa hollow-fiber filter module. OMV particles remain in the hollow fiber flow circuit, while 10 mL of undiluted OMV-depleted media, which passed through the filter pores, was collected. After collection of the OMV-depleted media, OMVs were eluted from the column with 10 mL of EMEM plus 10% FBS to maintain their initial concentration. Particles were quantified on a C400 microfluidic cartridge using a Spectradyne nCS1 instrument (Torrance, CA), which uses MRPS technology. Samples were diluted 1:20 in 1% PBS, and 5000 events were recorded. 100 µL OMVs or OMV-depleted media was added to the upper chamber of the transwell to evaluate their effect on TEER.

### Flow cytometry

Optical density was used as an approximation of bacteria concentration (OD 1 = 10^9^ bacteria/mL).^61^ Bacterial cultures were stained at room temperature for 30 mins with 15 µg/mL 4ʹ,6-Diamidino-2-Phenylindole, Dihydrochloride (DAPI) (Invitrogen, D1306) at 2.5 × 10^7^ bacteria/mL in 25 µL of PBS (Thermo Fisher Scientific, 70–013-032) supplemented with 1% bovine serum albumin (BSA) (Fisher Scientific, BP1600-100) and 0.05% azide (Sigma Aldrich, RTC000068-1 L).^61,62^ Plasma was added at a final dilution of 1:10 in 25 µL of PBS/BSA/azide in combination with fluorochrome-conjugated antibody (FITC-labeled mouse anti-human IgG clone G18-145 (BD, 555786)) for 30 mins in a U-bottom 96-well plate. Samples were resuspended in 200 µL of PBS/BSA/azide and analyzed on a LSR Fortessa (BD). The fluorescence threshold on the DAPI channel was set to 200 arbitrary units based on unstained bacteria for exclusive detection of fluorescently labeled bacteria. 110,000 events were collected, and data were analyzed using FlowJo software (version 10.7.2) (BD).

### Immunoblots

Protein expression was assessed via immunoblot for claudin 1 and claudin 4, using GAPDH as a loading control. Caco-2 BBe monolayers on transwell membranes were lysed with RIPA buffer (Thermo Fisher Scientific, 89900) and a protease inhibitor cocktail (Thermo Fisher Scientific, 78425). After centrifugation for 5 mins at 13,000 x g, the cleared lysate was denatured with 5X reducing buffer (Thermo Fisher Scientific, 39000) before heating the sample for 5 min at 95°C. 25 µL of lysate was loaded onto 0.75 mm 12% polyacrylamide gel and electrophoresed at 80 V until the pink dye reached the bottom. Wet transfer was performed for 1 h at 80 V onto PDVF membranes (Bio-Rad, Hercules, CA). The membrane was blocked with 5% milk and incubated with primary antibodies to claudin 1 (Sigma Aldrich, SAB4200534), claudin 4 (Abcam, Ab53156) or GAPDH (Sigma Aldrich, MAB374) overnight. Goat-anti rabbit (Sigma Aldrich, A4914-1ML) and goat-anti mouse (Bio-Rad, 170–6515) secondary antibodies were incubated for 1 h before imaging the membrane on an ImageQuant LAS 4000 (GE Healthcare, Chicago, IL). Images were exposed for 5 sec and digitalized for further quantification using Image Studio Lite (LI-COR Bioscience, Lincoln, NE).

### mRNA quantification

Caco-2 BBe monolayers on a transwell membrane were washed with PBS before extracting RNA using the Purelink RNA micro-scale kit (Thermo Fisher Scientific, 12183016) according to the manufacturer’s protocol, except as noted. The cell lysate was applied to the binding column twice to optimize RNA yield. Washes were performed as indicated in the manufacturer’s protocol and bound nucleic acid incubated with DNase (Thermo Fisher Scientific, 12185010) for 40 min before resuming with the remaining washes. RNA was eluted with 22 µL of ultrapure RNA/DNase free water and used to synthesize cDNA immediately after extraction with a High-Capacity RNA-to-cDNA Kit (Thermo Fisher Scientific, 4388950). RNA and cDNA were quantified on a Nanodrop spectrophotometer (Thermo Fisher Scientific). 100 ng of cDNA was added to the TaqMan (Thermo Fisher Scientific, 4331182) assay performed on a QuantStudio3 (Thermo Fisher Scientific) for the genes of interest: *CLDN*1, *CLDN*4, and *RPLPO* as an internal control (Thermo Fisher Scientific). Analysis used a 2^−ΔΔCt^ calculation to represent fold change relative to untreated cells.

### Statistical analysis

The unpaired t-test was used to analyze α- and β-diversity, microbial communities, and the content of fecal and plasma SCFAs. Spearman correlations were used for fecal and plasma SCFAs and immune mediator comparisons with the gut microbiota. Two-way ANOVA with multiple comparisons with Tukey correction was used for SCFAs, naloxone, OMV, and OMV-depleted media treatment and Sidak correction for *A. muciniphila* spent media, methadone, and combination treatment on barrier integrity. One-way ANOVA with multiple comparisons (Dunnett correction) was used for mRNA and protein expression levels. Graphical representations and statistical analysis were performed using PRISM v8.1.1 (GraphPad, San Diego, CA). All tests were 2-sided. A *p*-value less than 0.05 was considered statistically significant.

## Supplementary Material

Supplemental Material

## Figures and Tables

**Table:** 

ALI	Air-Liquid Interface
BHI	brain heart infusion
BSA	bovine serum albumin
CRP	C-reactive protein
DAMGO	[D-Ala^2^, N-Me-Phe^4^, Gly^5^-ol]-Enkephalin acetate salt
DAPI	4ʹ,6-Diamidino-2-Phenylindole, Dihydrochloride
DPDPE	[D-Pen 2,5] Enkephalin
EV	extracellular vesicles
GC-MS	gas chromatography-mass spectrometry
GI	gastrointestinal
HBV	Hepatitis B Virus
HCV	Hepatitis C Virus
HIV	Human immunodeficiency virus
IBD	Inflammatory Bowel Disease
IL-1β	Interleukin-1β
IL-6	Interleukin-6
IL-8	Interleukin-8
IFABP	Intestinal-type fatty acid-binding protein
LCN2	Lipocalin-2
LBP	Lipopolysaccharide binding protein
MIP1α	macrophage inflammatory protein 1 α
MMT	methadone maintenance treatment
MRPS	microfluidic resistive pulse sensing
ns	not significant
OIBD	opioid-induced bowel dysfunction
OR	opioid receptors
OD	optical density
OMV	outer membrane vesicles
PCA	Principal component analysis
SCFAs	short chain fatty acids
TFF	tangential flow filtration
TJ	tight junctions
TEER	transepithelial electrical resistance
TNFα	tumor necrosis factor α.
