# Supplementary material for: Chronic opioid use modulates human enteric microbiota and intestinal barrier integrity
Source: Gut Microbes. 2021 Jul 27;13(1):1946368. doi: 10.1080/19490976.2021.1946368 (PMC8317955; doi:10.1080/19490976.2021.1946368)
Supplement: Supplemental Material [file KGMI_A_1946368_SM2769.zip › supplementary/Gut microbes Supplemental info v9.docx]

**SUPPLEMENTAL INFORMATION**

**Supplemental Table 1. Self-reported information from study population survey**

| **Demographics** | **Non-opioid users** | **Methadone** |
| --- | --- | --- |
| Opioid type (range of duration)   - Short (≤5 years) - Long (≥10 years) | N/A | Methadone (1.5-18 years)  4  4 |
| Comorbidities   - HIV - HCV - HBV | 0  0  0 | 0  4  0 |
| **Medications** | | |
| Antibiotic use | Amoxicillin (1 donor)  Doxycycline (1 donor) | Clindamycin (1 donor)  Azithromycin (1 donor) |
| Diarrhea (Y/N/Not reported) | 3/11/14 | 3/12/19 |
| Constipation (Y/N/Not reported) | 2/12/14 | 7/8/19 |

**Supplemental Table 2. Plasma Immune mediators**

| **Immune mediator** | **Non-opioid users Average / (range)** | **Methadone Average / (range)** | **p-value** |
| --- | --- | --- | --- |
| **IL-6 (pg/mL)** | **0.98 / (0.05-5.1)** | **2.65 / (0.10-11.5)** | **0.03** |
| **TNFα (pg/mL)** | **4.67 / (3.34-6.63)** | **6.85 / (3.20-14.92)** | **0.01** |
| CRP (ng/mL) | 206.74 / (0.4-2375) | 25.01 / (0.3-51.6) | 0.26 |
| LBP (ng/mL) | 24.05 / (11.5-35.3) | 24.96 / (0.7-34.8) | 0.83 |
| IFABP (pg/mL) | 68.64/ (01.46-187) | 57.98 / (11.8-120) | 0.64 |
| MIP1α/CCL3 (pg/mL) | 0.07 / (0-0.94) | 0.52 / (0-6.66) | 0.36 |
| IL-1β/IL-1F2 (pg/mL) | 0.09 / (0-0.70) | 0.09 / (0-1.18) | 0.96 |
| IL-8 | 0 / (0) | 19.92 / (0-418.48) | 0.41 |
| LCN2 (ng/mL) | 2.98 / (0.46-7.16) | 4.59 / (0.17-17.73) | 0.19 |

Bold text reached statistical significance, using a two-tailed t-test.

**Supplemental Table 3. Statistical table of multiple comparisons (Figure 5 panel c)**

| **3 hours** |  |  |
| --- | --- | --- |
| Methadone (100µM) + Naloxone (100µM) *vs.* Naloxone (10uM) | *** | 0.0003 |
| Methadone (100µM) + Naloxone (100µM) *vs.* Naloxone (1uM) | *** | 0.0006 |
| **6 hours** |  |  |
| Methadone (100uM) *vs.* Methadone (100µM) + Naloxone (100µM) | * | 0.0338 |
| Methadone (100µM) + Naloxone (100µM) *vs.* Naloxone (100uM) | ** | 0.0091 |
| Methadone (100µM) + Naloxone (100µM) *vs.* Naloxone (10uM) | **** | <0.0001 |
| Methadone (100µM) + Naloxone (100µM) *vs.* Naloxone (1uM) | **** | <0.0001 |
| **24 hours** |  |  |
| Methadone (100uM) *vs.* Methadone (100µM) + Naloxone (100µM) | **** | <0.0001 |
| Methadone (100uM) *vs.* Naloxone (100uM) | **** | <0.0001 |
| Methadone (100uM) *vs.* Naloxone (10uM) | **** | <0.0001 |
| Methadone (100uM) *vs.* Naloxone (1uM) | **** | <0.0001 |
| Methadone (100µM) + Naloxone (100µM) *vs.* Methadone (100µM) + Naloxone (10µM) | *** | 0.0004 |
| Methadone (100µM) + Naloxone (100µM) *vs.* Methadone (100µM) + Naloxone (1µM) | ** | 0.0017 |
| Methadone (100µM) + Naloxone (100µM) *vs.* Naloxone (100uM) | **** | <0.0001 |
| Methadone (100µM) + Naloxone (100µM) *vs.* Naloxone (10uM) | **** | <0.0001 |
| Methadone (100µM) + Naloxone (100µM) *vs.* Naloxone (1uM) | **** | <0.0001 |
| Methadone (100µM) + Naloxone (10µM) *vs.* Naloxone (100uM) | **** | <0.0001 |
| Methadone (100µM) + Naloxone (10µM) *vs.* Naloxone (10uM) | **** | <0.0001 |
| Methadone (100µM) + Naloxone (10µM) *vs.* Naloxone (1uM) | **** | <0.0001 |
| Methadone (100µM) + Naloxone (1µM) *vs.* Naloxone (100uM) | **** | <0.0001 |
| Methadone (100µM) + Naloxone (1µM) *vs.* Naloxone (10uM) | **** | <0.0001 |
| Methadone (100µM) + Naloxone (1µM) *vs.* Naloxone (1uM) | **** | <0.0001 |
| **48 hours** |  |  |
| Methadone (100uM) *vs.* Methadone (100µM) + Naloxone (100µM) | **** | <0.0001 |
| Methadone (100uM) *vs.* Naloxone (100uM) | **** | <0.0001 |
| Methadone (100uM) *vs.* Naloxone (10uM) | **** | <0.0001 |
| Methadone (100uM) *vs.* Naloxone (1uM) | **** | <0.0001 |
| Methadone (100µM) + Naloxone (100µM) *vs.* Methadone (100µM) + Naloxone (10µM) | **** | <0.0001 |
| Methadone (100µM) + Naloxone (100µM) *vs.* Methadone (100µM) + Naloxone (1µM) | *** | 0.0002 |
| Methadone (100µM) + Naloxone (100µM) *vs.* Naloxone (100uM) | **** | <0.0001 |
| Methadone (100µM) + Naloxone (100µM) *vs.* Naloxone (10uM) | **** | <0.0001 |
| Methadone (100µM) + Naloxone (100µM) *vs.* Naloxone (1uM) | **** | <0.0001 |
| Methadone (100µM) + Naloxone (10µM) *vs.* Naloxone (100uM) | **** | <0.0001 |
| Methadone (100µM) + Naloxone (10µM) *vs.* Naloxone (10uM) | **** | <0.0001 |
| Methadone (100µM) + Naloxone (10µM) *vs.* Naloxone (1uM) | **** | <0.0001 |
| Methadone (100µM) + Naloxone (1µM) *vs.* Naloxone (100uM) | **** | <0.0001 |
| Methadone (100µM) + Naloxone (1µM) *vs.* Naloxone (10uM) | **** | <0.0001 |
| Methadone (100µM) + Naloxone (1µM) *vs.* Naloxone (1uM) | **** | <0.0001 |

p ≤0.05 (*), p ≤0.005 (**), p ≤0.0005 (***), p ≤0.0001 (****)

**Supplemental Table 4. Parent to daughter ion transitions**

| **Acids** | **Parent to daughter ion transitions** |
| --- | --- |
| **Acetic** | m/z 61.0 → 43.0 for acetic acid, m/z 63.0 → 45.0 for [13C2]-acetic acid |
| **Butyric** | m/z 61.0 → 43.0 m/z 71.0 → 41.0 for butyric acid, m/z 78.1 → 46.1 for D7-butyric acid |
| **Isovaleric** | m/z 85.1 → 57.1 for isovaleric acid, m/z 87.1 → 59.1 for D2-isovaleric acid |
| **Lactic** | m/z 135.1 → 45.1 for lactic acid, m/z 138.1 → 48.0 for D3-lactic acid |
| **Propionic** | m/z 75.1 → 57.0 for propionic acid, m/z 77.1 → 59.0 for D2-propionic acid |
| **Succinic** | m/z 101.1 → 55.0 for succinic acid, m/z 105.1 → 57.0 for D6-succinic acid |

**Supplemental Figure 1. Dysbiosis of the core microbiota varies with duration of methadone treatment.**

The relative abundance of the core microbiota, *Bacteroidetes*, *Firmicutes*, *Actinobacteria*, and *Proteobacteria* was compared based on the duration of methadone treatment. Those study volunteers who self-reported the duration of their methadone treatment (8 of 34) were grouped as short (≤5 years) (blue) and long (≥10 years) (red) with 4 individuals in each group. The relative abundance of *Bacteroidetes* (p<0.0001) was significantly greater in the long-term cohort (red) compared to a short duration (blue) of methadone treatment. The opposite was observed for *Firmicutes* (p=0.0002), where the short term (blue) cohort had elevated levels of this phylum compared to the long-term cohort (red). Abundance of *Actinobacteria* (p=0.07) and *Proteobacteria* (p=0.24) revealed no dependence on the duration of methadone treatment.

**Supplemental Figure 2. Core microbiota, plasma and fecal SCFAs, and plasma immune mediators do not correlate with methadone use.**

**a,** Gas chromatography-Mass spectrometry was used to calculate the content of short chain fatty acids in plasma. Propionate, but not acetate or butyrate, is elevated in treated individuals compared to non-opioid users (p=0.05).

**b,** A Spearman correlation between plasma SCFAs and the relative abundance of the core bacteria from non-opioid users and methadone-treated individuals. Heatmap shows Spearman r coefficient values, and asterisks denotate statistical significance. Acetate correlates negatively with *Bacteroidetes* (p=0.008) and positively with *Firmicutes* (p=0.03) in methadone-treated individuals. Butyrate correlates positively with *Firmicutes* (p=0.04) in methadone-treated individuals. p ≤0.05 (*), p ≤0.005 (**).

**c, d, e, Spearman** correlation between immune mediators and the core microbiota **(c)**, fecal SCFAs **(d)**, and plasma SCFAs **(e)** from non-opioid users and methadone-treated individuals. Heatmap shows Spearman r coefficient values, asterisks denotate statistical significance. ND (not determined) was used when correlations could not be calculated due to no measurable IL-8 levels. Lipocalin 2 levels from methadone-treated individuals showed a positive correlation with *Verrucomicrobia* (p=0.04) and a negative correlation with *Bacteroidetes* (p=0.04). Non-opioid users showed a negative correlation between *Actinobacteria* and *Bacteroidetes* and LBP (p=0.04) and CRP (p=0.01), respectively. In non-opioid users a positive correlation between *Firmicutes* and IL-6 (p=0.04) and CRP (p=0.001) was observed, while a negative correlation between *Verrucomicrobia* and I-FABP (p=0.03) was seen. TNFα levels from non-opioid users correlated positively with *Verrucomicrobia* (p=0.01). Non-opioid users showed a positive correlation between fecal acetate, propionate, and butyrate with IL-6 (p=0.006, 0.005, 0.008) and CRP (p=0.01,0.004, 0.05), respectively. I-FABP showed a positive correlation with plasma butyrate (p=0.03) in non-opioid users. Negative correlations were observed between plasma acetate (p=0.01) and butyrate (p=0.01) and MIP1α in methadone-treated individuals.

**Supplemental Figure 3. SCFAs and *Akkermansia muciniphila* components from spent media on paracellular permeability.**

**a,** Exogenous SCFAs were added to the upper chamber of the transwell and TEER followed over time. **b,** Tangential flow filtration used to isolate *A. muciniphila* OMV-depleted media and OMVs. **c,** Particle concentration as determined using MRPS technique (nCS1).

**Supplemental Figure 4. Methadone stimulation of an increase in TEER is dose dependent.**

Graded concentrations of methadone were added in the lower chamber of a Caco-2 BBe monolayer transwell culture. TEER was measured at 3, 6, 24, 48, and 72 h.
