# Supplementary figures and images for: Chronic opioid use modulates human enteric microbiota and intestinal barrier integrity
Source: Gut Microbes. 2021 Jul 27;13(1):1946368. doi: 10.1080/19490976.2021.1946368 (PMC8317955; doi:10.1080/19490976.2021.1946368)

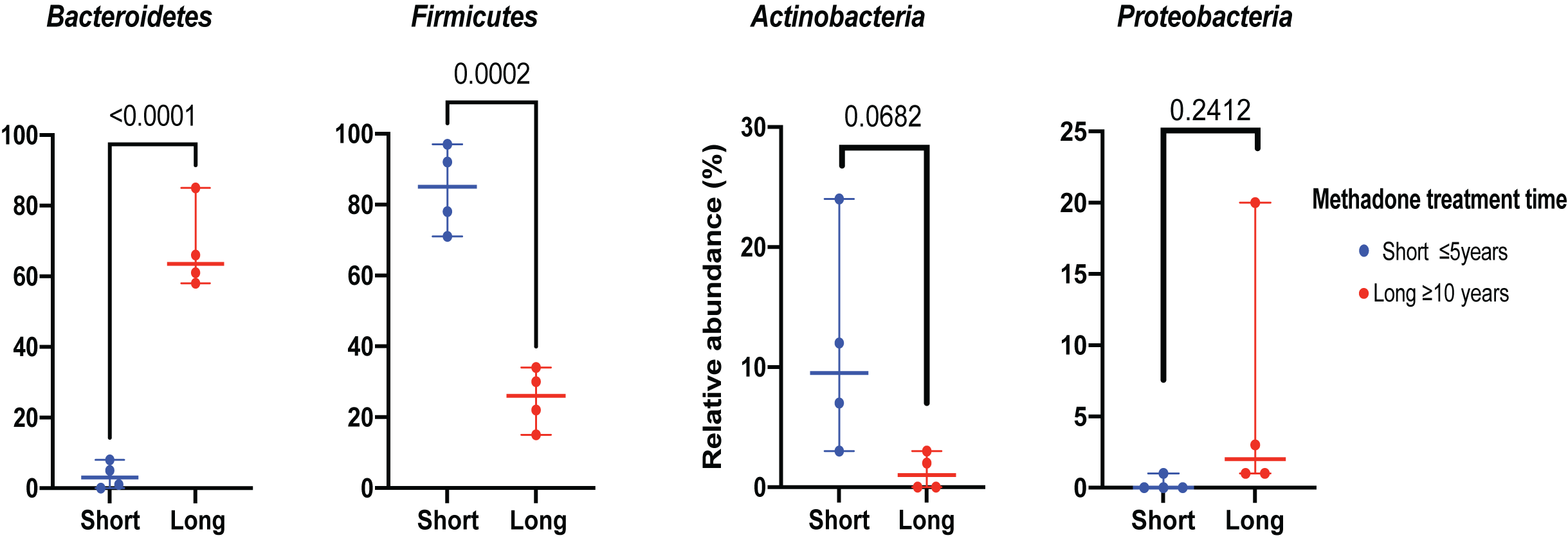

Supplement: Supplemental Material [file KGMI_A_1946368_SM2769.zip › supplementary/SF1 .tif]

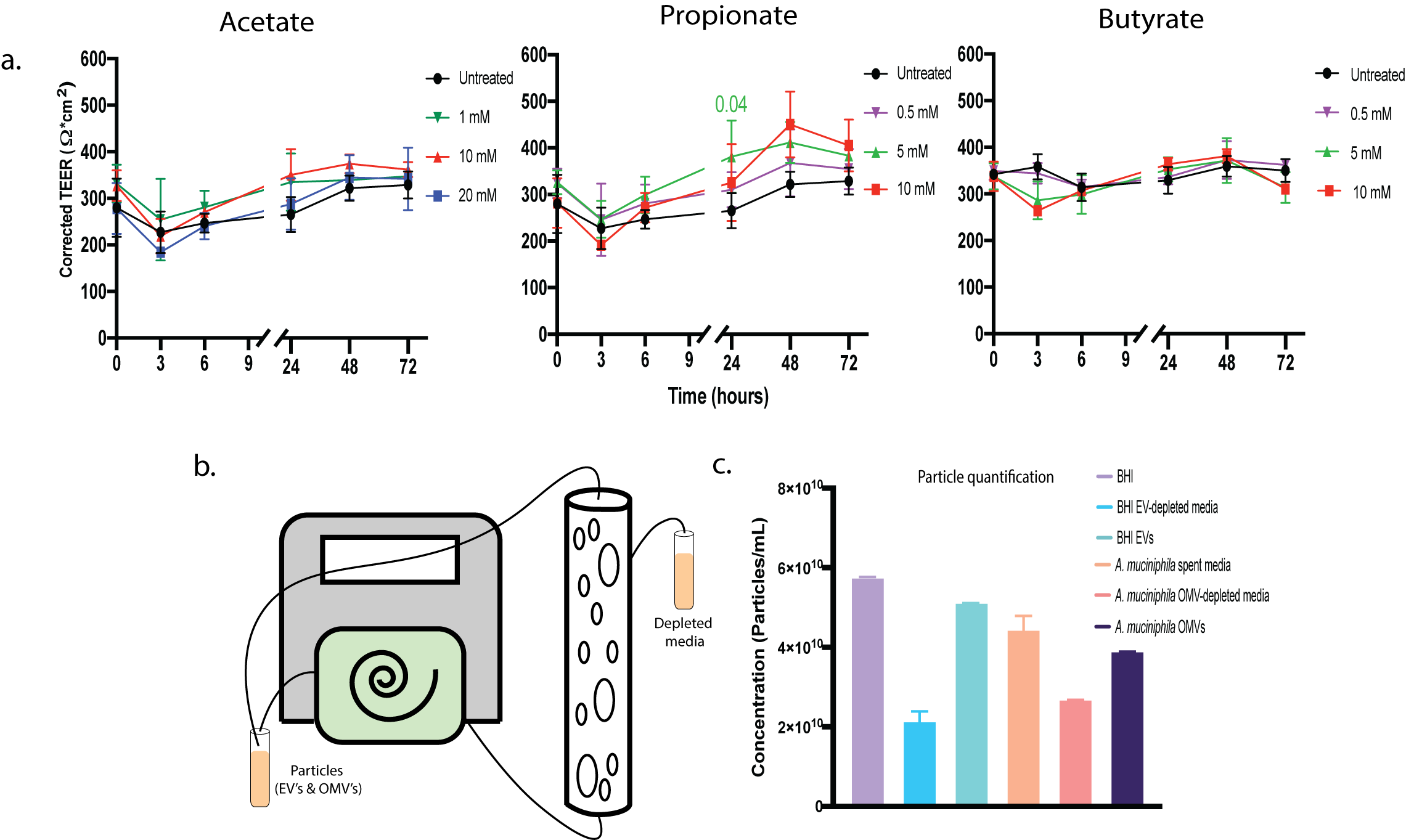

Supplement: Supplemental Material [file KGMI_A_1946368_SM2769.zip › supplementary/SF3 .tif]

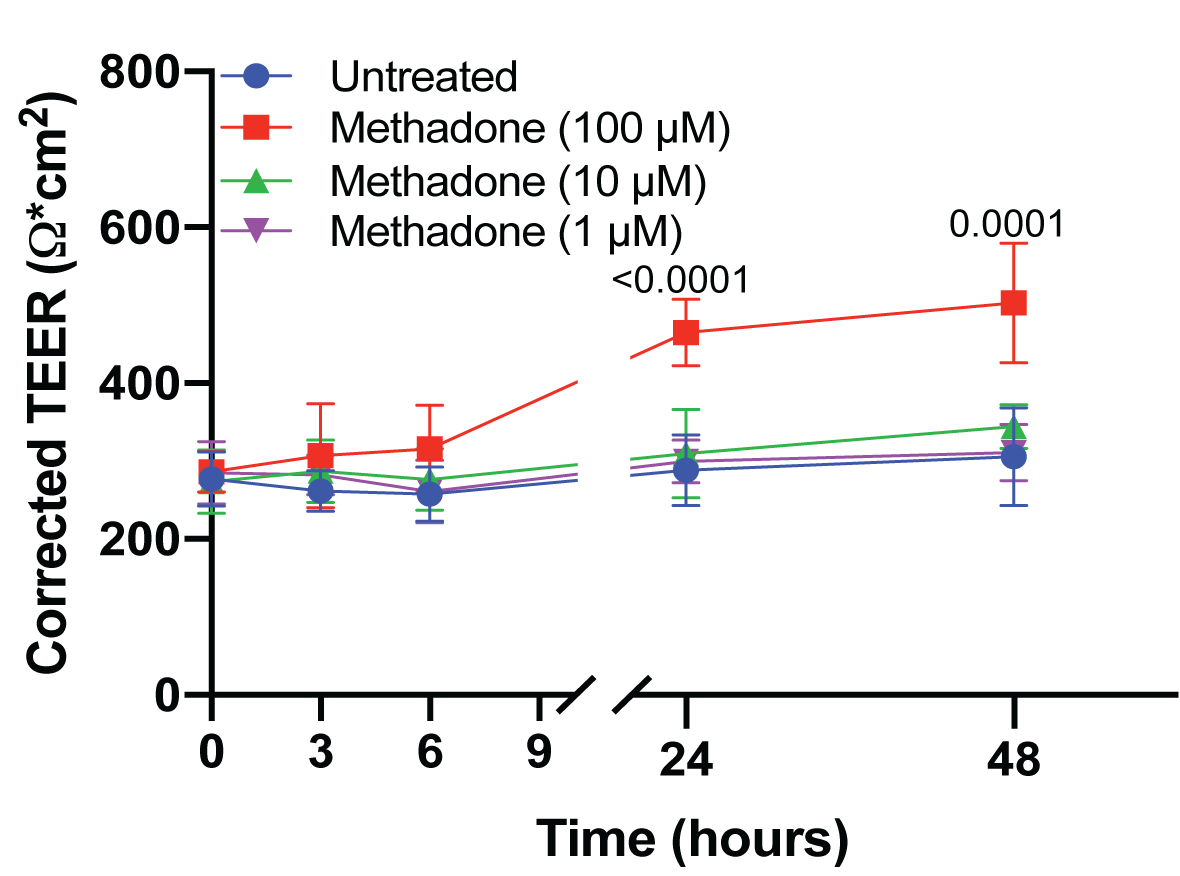

Supplement: Supplemental Material [file KGMI_A_1946368_SM2769.zip › supplementary/SF4 .tif]
